# Cardiac Magnetic Resonance to Predict Cardiac Mass Malignancy: The CMR Mass Score

**DOI:** 10.1161/CIRCIMAGING.123.016115

**Published:** 2024-03-19

**Authors:** Pasquale Paolisso, Luca Bergamaschi, Francesco Angeli, Marta Belmonte, Alberto Foà, Lisa Canton, Damiano Fedele, Matteo Armillotta, Angelo Sansonetti, Francesca Bodega, Sara Amicone, Nicole Suma, Emanuele Gallinoro, Domenico Attinà, Fabio Niro, Paola Rucci, Elisa Gherbesi, Stefano Carugo, Saima Mushtaq, Andrea Baggiano, Anna Giulia Pavon, Marco Guglielmo, Edoardo Conte, Daniele Andreini, Gianluca Pontone, Luigi Lovato, Carmine Pizzi

**Affiliations:** 1 Clinical Cardiology and Cardiovascular Imaging Unit, Galeazzi-Sant’Ambrogio Hospital, IRCCS, Milan, Italy (P.P., E. Gallinoro, E.C., D. Andreini).; 2 Department of Biomedical and Clinical Sciences (P.P., E. Gallinoro, E.C., D. Andreini), University of Milan, Italy.; 3 Department of Clinical Sciences and Community Health (E. Gherbesi, S.C., A.B., G.P.), University of Milan, Italy.; 4 Department of Biomedical, Surgical and Dentals Sciences (G.P.), University of Milan, Italy.; 5 Department of Advanced Biomedical Sciences, University of Naples, Federico II, Italy (P.P., M.B.).; 6 Cardiology Unit, Cardiac Thoracic and Vascular Department, IRCCS Azienda Ospedaliera-Universitaria di Bologna (L.B., F.A., A.F., L.C., D.F., M.A., A.S., F.B., S.A., N.S., C.P.).; 7 Department of Medical and Surgical Sciences, DIMEC (L.B., F.A., A.F., L.C., D.F., M.A., A.S., F.B., S.A., N.S., C.P.).; 8 Alma Mater Studiorum (L.B., F.A., A.F., L.C., D.F., M.A., A.S., F.B., S.A., N.S., C.P.), University of Bologna, Italy.; 9 Division of Hygiene and Biostatistics, Department of Biomedical and Neuromotor Sciences, Alma Mater Studiorum (P.R.), University of Bologna, Italy.; 10 Cardiovascular Center Aalst, OLV Hospital, Aalst, Belgium (M.B.).; 15 Pediatric and Adult CardioThoracic and Vascular, Oncohematologic and Emergency Radiology Unit, IRCCS Azienda Ospedaliero-Universitaria di Bologna, Bologna, Italy (D. Attinà, F.N., L.L.).; 11 Department of Cardio-Thoracic-Vascular Diseases, Foundation IRCCS Ca’ Granda Ospedale Maggiore Policlinico, Milan, Italy (E. Gherbesi, S.C.).; 12 Department of Perioperative Cardiology and Cardiovascular Imaging, Centro Cardiologico Monzino IRCCS, Milan, Italy (S.M., A.B.).; 13 Division of Cardiology, Cardiocentro Ticino Institute, Ente Ospedaliero Cantonale, Lugano, Switzerland (A.G.P.).; 14 Department of Cardiology, Division of Heart and Lungs, Utrecht University, Utrecht University Medical Center, the Netherlands (M.G.).

**Keywords:** cardiac magnetic resonance (CMR), cardiac masses, echocardiography, prognosis

## Abstract

**BACKGROUND:**

Multimodality imaging is currently suggested for the noninvasive diagnosis of cardiac masses. The identification of cardiac masses’ malignant nature is essential to guide proper treatment. We aimed to develop a cardiac magnetic resonance (CMR)-derived model including mass localization, morphology, and tissue characterization to predict malignancy (with histology as gold standard), to compare its accuracy versus the diagnostic echocardiographic mass score, and to evaluate its prognostic ability.

**METHODS:**

Observational cohort study of 167 consecutive patients undergoing comprehensive echocardiogram and CMR within 1-month time interval for suspected cardiac mass. A definitive diagnosis was achieved by histological examination or, in the case of cardiac thrombi, by histology or radiological resolution after adequate anticoagulation treatment. Logistic regression was performed to assess CMR-derived independent predictors of malignancy, which were included in a predictive model to derive the CMR mass score. Kaplan-Meier curves and Cox regression were used to investigate the prognostic ability of predictors.

**RESULTS:**

In CMR, mass morphological features (non-left localization, sessile, polylobate, inhomogeneity, infiltration, and pericardial effusion) and mass tissue characterization features (first-pass perfusion and heterogeneity enhancement) were independent predictors of malignancy. The CMR mass score (range, 0–8 and cutoff, ≥5), including sessile appearance, polylobate shape, infiltration, pericardial effusion, first-pass contrast perfusion, and heterogeneity enhancement, showed excellent accuracy in predicting malignancy (areas under the curve, 0.976 [95% CI, 0.96–0.99]), significantly higher than diagnostic echocardiographic mass score (areas under the curve, 0.932; *P*=0.040). The agreement between the diagnostic echocardiographic mass and CMR mass scores was good (κ=0.66). A CMR mass score of ≥5 predicted a higher risk of all-cause death (*P*<0.001; hazard ratio, 5.70) at follow-up.

**CONCLUSIONS:**

A CMR-derived model, including mass morphology and tissue characterization, showed excellent accuracy, superior to echocardiography, in predicting cardiac masses malignancy, with prognostic implications.

**CLINICAL PERSPECTIVE**

In this article, we outlined the pivotal role of cardiac magnetic resonance (CMR) in the diagnostic work-up of patients with cardiac tumors, either benign or malignant. Compared with echocardiography, which might already inform on the possible malignant nature of cardiac masses using a dedicated score, CMR has the unique advantage of providing tissue characterization in a noninvasive setting. In this regard, we showed that CMR-derived mass morphological features (non-left localization, sessile, polylobate, inhomogeneity, infiltration, and pericardial effusion) and mass tissue characterization features (first-pass perfusion and heterogeneity enhancement) were independent predictors of malignancy. When combining these variables in a weighted score, the CMR mass score (range, 0–8 and cutoff, ≥5), the predictive yield for malignancy was excellent (areas under the curve, 0.976 [95% CI, 0.96–0.99]). In addition, the CMR mass score also had prognostic implications, with values of ≥5 predicting a higher risk of all-cause death (hazard ratio, 5.70; *P*<0.001) at follow-up. Thus, the CMR mass score might be a reliable, easily applicable tool to characterize cardiac masses, and to predict the malignant nature with high accuracy. Multimodality imaging, including at least echocardiography and CMR, should be recommended in the diagnostic work-up of cardiac mass.

**See Editorial by Hoit**

During the last decades, a significant change in the diagnostic work-up of cardiac masses (CMs) has occurred due to the introduction and wider availability of noninvasive imaging techniques. Although histology remains the gold standard for the definite diagnosis of malignancy, multimodality imaging approaches (including echocardiography, cardiac magnetic resonance [CMR], cardiac computed tomography, and positron emission tomography) are increasingly being used for the diagnosis of CMs.^1–3^ Echocardiography is recognized as the first-line test, and CMR the most comprehensive technique. The correct identification of CMs’ nature is essential to guide the proper treatment.^4–7^

Echocardiography provides crucial information about CMs, including location, morphology, pericardial involvement, and hemodynamic impact.^3,8^ We showed that easily assessable echocardiographic parameters (ie, infiltration, polylobate shape, presence of moderate/severe pericardial effusion, sessile appearance, inhomogeneity, and non-left localization) are independent predictors of malignancy, confirmed at histology. The integration of those predictors into a multiparametric score (the diagnostic echocardiographic mass [DEM] score) led to a significant increase in the diagnostic accuracy in predicting malignancy.^9^ This was confirmed both for the weighted and the unweighted score.^10^ However, the accuracy of echocardiography could be affected by a poor acoustic window, presence of artifacts, and operator variability and expertise.

Compared with echocardiography, CMR allows for high spatial resolution and multiplanar imaging, comprehensively characterizing mass morphology, tissue characterization, blood perfusion, and extracardiac findings.^11–13^ Several studies have reported a remarkable accuracy of CMR in CMs characterization compared with histology.^12,14,15^ However, they are limited due to the small sample size, the underrepresentation of some CMs histotypes, and the lack of a direct comparison between echocardiography and CMR.

In the present study, in a broad cohort of patients with CMs, we aimed: (1) to assess the accuracy of the previously validated DEM score applied to CMR; (2) to evaluate the incremental value of tissue characterization in CMR, when integrated into the models to predict malignancy; (3) to evaluate the agreement between echocardiography and CMR in identifying morphological features of CMs associated with malignancy; (4) to prove the prognostic ability of the CMR-derived model with the highest performance.

## METHODS

The data that support the findings of this study are available from the corresponding author upon reasonable request.

### Study Population

This observational cohort study included all consecutive patients undergoing echocardiography and CMR, within 1-month time interval, for suspected CM at University Hospital Policlinico Sant’Orsola Malpighi, in Bologna, Italy, from January 2004 to December 2022. A definitive diagnosis was achieved in all cases by the histological examination of biopsy/surgical samples or, in the case of cardiac thrombi, by histology or radiological resolution after adequate anticoagulation treatment. After the diagnostic work-up, CMs were classified as benign or malignant. Benign masses included pseudotumors and primary cardiac benign tumors, whereas malignant masses comprised primary cardiac malignant and secondary tumors.^16^ Further details on the definition and classifications of CM are provided in Supplemental Material, Extended Methods. The study flow chart is shown in Figure S1. All patients were managed according to the Declaration of Helsinki and provided informed consent for the anonymous publication of scientific data. The study protocol was approved by the local Ethics Committee (Registration No. 102/2017/Oss/AOUBo).

### Echocardiography

All patients were evaluated by a comprehensive echocardiogram following the recommendations of the American Society of Echocardiography and the European Association of Cardiovascular Imaging.^17–20^ After the acquisition, 2 cardiologists, experts in echocardiography (P. Paolisso and L. Bergamaschi), blinded to clinical information and CM histology, analyzed the echocardiographic exams and completed a prespecified worksheet assessing the morphological mass features, as described previously.^9,10^ A third echocardiography cardiologist solved disagreements in imaging evaluation. Details about the echocardiographic protocol and the definition of each assessed CMs’ features are reported in the Supplemental Material, Extended Methods.

### Cardiac Magnetic Resonance

The examinations were performed on a 1.5 T CMR scanner (Ingenia; Philips Healthcare, Best, the Netherlands). All CMR investigations were analyzed in consensus by qualified radiologists with ≥10 years of experience in CMR (L. Lovato and F. Niro), blinded to patient data and clinical reports. CMR analysis was performed using commercially available software (Circle CVI: cvi42) and aimed to evaluate: (1) mass localization; (2) mass morphology (including assessment of infiltration); (3) mass tissue characterization precontrast and postcontrast; (4) mass vascularization; (5) functional and dimensional assessment of the cardiac chambers. The following sequences were obtained: (1) ECG-gated balanced steady-state free precession pulse sequence for cine images; (2) black blood T1- and T2-weighted images; (3) black blood T1-weighted fat-suppressed (FAT-SAT), when required; (4) first-pass perfusion; segmented phase-sensitive inversion recovery sequences, used to detect (5) early gadolinium enhancement (EGE), and (6) late gadolinium enhancement (LGE), respectively.^21^ Additional information about the CMR acquisition protocol is specified in the Supplemental Material, Extended Methods.

**Figure 1.**
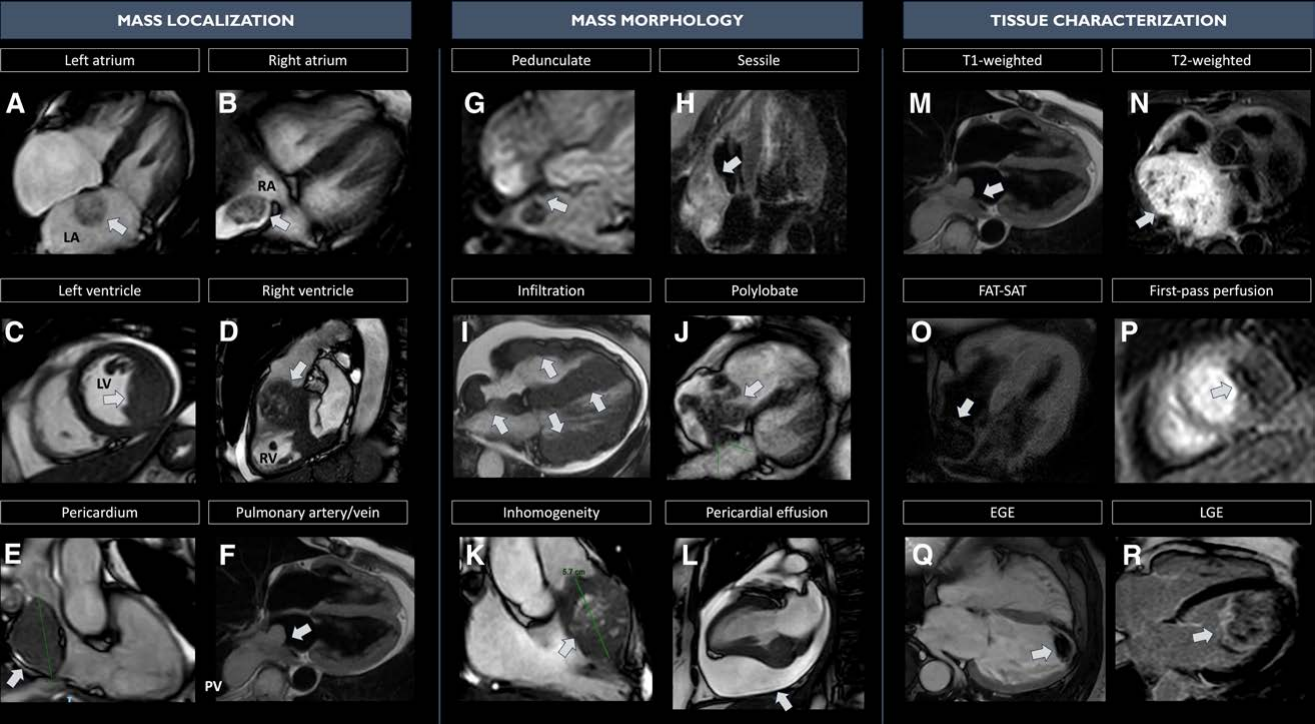
**Case examples of cardiac masses features assessed at cardiac magnetic resonance (CMR) through a systematic approach.** Cardiac masses (CMs) assessment at CMR should aim (at least) to evaluate mass localization, mass morphology, and tissue characterization. (I) Mass localization (**A** through **F**): examples of the most common localization of CMs (left atrium [myxoma], right atrium [neoplastic thrombus in patient with renal carcinoma], left ventricle [metastasis of melanoma], right ventricle [metastasis of urothelial carcinoma], pericardium [pericardial angioma], pulmonary artery/vein [atrial leiomyosarcoma]). (II) Mass morphology (**G** through **L**): examples of the CMs’ morphological features assessed (pedunculate [atrial myxoma], sessile [cardiac angiosarcoma], infiltration [aggressive B Lymphoma], polylobate [metastasis of hepatocellular carcinoma], inhomogeneous appearance [metastasis of urothelial carcinoma], pericardial effusion [metastasis of melanoma]). (III) Mass tissue characterization (**M** through **R**): Hypo/iso/hyperintensity at the following sequences: T1-weighted (atrial leiomyosarcoma), T2-weighted (pericardial paraganglioma), T1, fat saturation (FAT-SAT; lipoma of the right atrium), first-pass perfusion (metastasis of melanoma), early gadolinium enhancement (EGE; left ventricular thrombus), late gadolinium enhancement (LGE; metastasis of melanoma). T1w-TSE indicates T1-weighted turbo spin echo; and T2w-TSE, T2-weighted turbo spin echo.

After CMR analysis, the cardiac imager filled a prespecified 3-section worksheet assessing the following mass features: (1) mass location (left/right, atrium/ventricle, pericardium, great vessels), site of attachment (interatrial/interventricular septum or roof/side wall of the atrium, ventricular free wall); (2) mass morphology: dimension, shape (regular/irregular; sessile—attached directly by the base and not raised upon a stalk, pedunculated—raised upon a stalk—or polylobate—having 2 or more lobes), margins (well defined/irregular – whether >50% of the border was clearly demarcated), mobility, inhomogeneous appearance (assessed in at least one among cine, T1 or T2 weighted images), infiltration (defined as at least one between: (1) disruption of neighboring tissue and extension of the mass across the pericardium into myocardium, with interruption of epicardial and endocardial contours, (2) increased thickness in comparison with the adjacent myocardial segments, determining hypo/dys/akinesia of a focal myocardial area compared with closest cardiac segments in absence of coronary distribution that could lead to the suspicion of ischemic etiology), (3) mass tissue characterization as defined by qualitative intensity pattern (hypointense, isointense, or hyperintense as compared with normal myocardium) and signal intensity ratio, defined as the signal intensity of the mass relative to the adjacent, uninvolved myocardium,^11,21^ presence or absence of contrast first-pass perfusion, EGE or LGE in the mass, (4) presence and severity of pericardial effusion. Case examples are provided in Figure 1.

### Statistical Analysis

The normality of the distribution of continuous variables was assessed using the Shapiro-Wilks test. Continuous variables with a normal distribution were expressed as the mean ± SD and nonnormally distributed variables as the median and interquartile range. Categorical variables were expressed as counts and percentages. Comparison of continuous data between groups was performed using Student *t* test or Mann-Whitney *U* test, as appropriate. Categorical data were compared between groups using the χ^2^ test or Fisher exact test as appropriate. CMR-derived features related to mass localization, morphology, and tissue characterization that were potentially predictive of malignancy were included in univariable logistic regression analyses. Variables showing statistical significance at the 10% level in univariable logistic regression were selected for multivariable logistic regression analysis, avoiding combinations of variables that would lead to collinearity, tested using the variance inflation factor. Two different CMR-derived models to predict malignancy were tested. First, we assessed the predictive accuracy of the CMR-derived model, including mass localization and morphology, using the variables included in the previously validated DEM score (Model 1, including infiltration, polylobate mass, pericardial effusion, sessile, inhomogeneity, and non-left localization).^9^ Second, we tested the incremental value of tissue characterization features when added to the mass localization+morphology model. Among the mass tissue characterization variables, those showing a statistically significant association with malignancy at univariate analysis were entered into a multivariate model. Variables predicting malignancy independently in the multivariable logistic regression analysis were used to build the predictive score and were integrated into Model 2 (CMR mass score, including infiltration, polylobate mass, pericardial effusion, sessile, first-pass contrast perfusion, heterogeneity enhancement). Specifically, the regression coefficient of each of these variables was divided by the smallest coefficient in the model and allocated a weight accordingly. The overall risk score was obtained by summing the weights thereby obtained from all coefficients. Cutoffs for all points of the scores were created. For each cutoff, accuracy indicators of malignancy were calculated. The best cutoff to predict malignancy was determined as the one that maximized Youden index (sensitivity+specificity-1). To evaluate and compare the accuracy of CMR models in predicting malignancy, the areas under the curve (AUCs) were calculated and compared using DeLong test. The agreement between echocardiographic and CMR-derived models was tested using accuracy indicators (sensitivity, specificity, positive predictive value, negative predictive value, and Cohen’s Kappa coefficient). A sensitivity analysis was conducted testing the diagnostic performance of the CMR mass score in the study population excluding patients with LV thrombus, being clinical evaluation and echocardiography often enough to achieve a correct diagnosis of LV thrombus. The interoperator variability in CMR mass score assessment was tested by Cohen’s Kappa (κ), with κ≥0.70 denoting adequate agreement. Finally, Kaplan-Meier curves were estimated to assess the prognostic ability of the CMR mass score to predict patient survival. The significance level was set at *P*<0.05. Statistical analyses were performed using R statistical software version 4.2.1 (R Foundation for Statistical Computing, Vienna, Austria), Statistical Package for Social Sciences, v28.0 (SPSS, PC version, Chicago, IL), and GraphPad Prism (GraphPad Software Inc., CA).

## RESULTS

### Study Population

A total of 167 patients included in the Bologna Cardiac Masses Registry underwent complete echocardiographic and CMR evaluation, within 1 month, from January 2004 to December 2022 (Figure S1). The median time between echocardiography and CMR was 8 (4–22) days. Sixteen CMs [4 benign masses (ie, 3 thrombi, and 1 paraganglioma) and 12 malignant (ie, 7 primary cardiac tumors and 5 metastasis)] were missed by the echocardiographic assessment and visualized only by CMR. Thus, the diagnostic accuracy of echocardiography in detecting a CM was 151/167 (90.4%). Based on histological examination, we identified 94 (56.3%) patients with benign CMs and 73 (43.7%) with malignancies. Histological characterization of benign and malignant masses is reported in Table 1. Each patient enrolled completed the follow-up, with a median follow-up time of 14 months and a maximum of 60 months (5 years). The baseline characteristics, clinical presentation, and echocardiographic findings stratified by CM malignancy are reported in Supplemental Material, Extended Results; Tables S1 and S2.

**Table 1.**
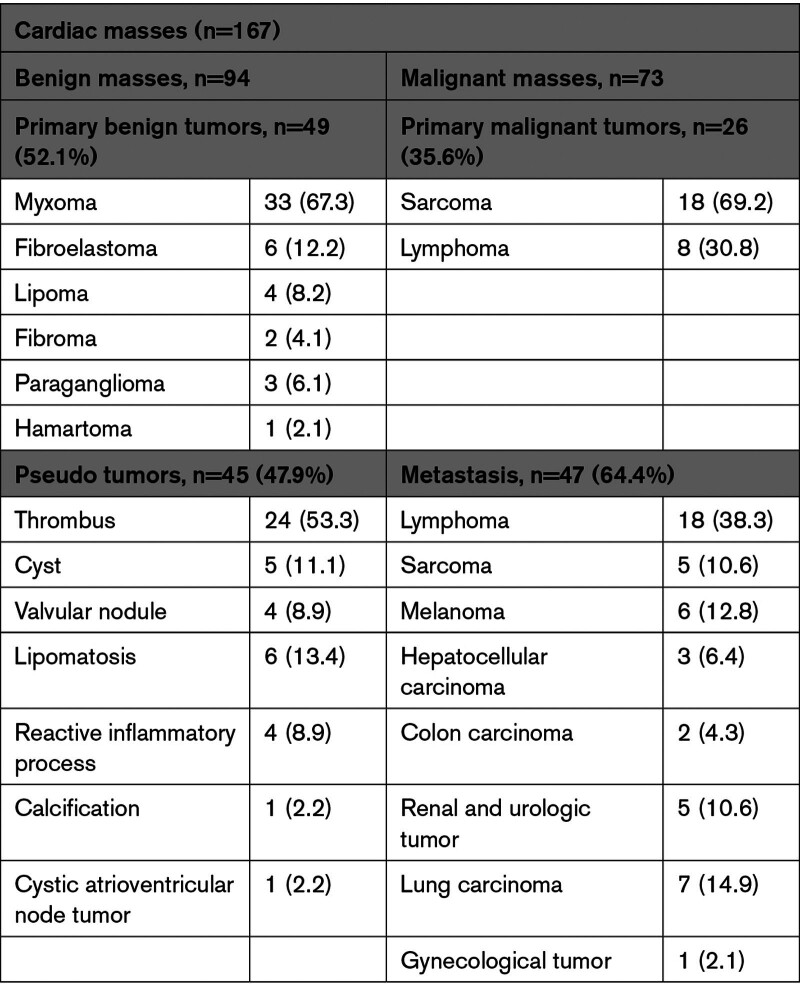
Histological Characterization of Benign and Malignant Masses of Study Population

### Benign Versus Malignant Masses: CMR Features

CMR features significantly differed between benign and malignant masses (Table 2). Benign formations were often located in the left heart chambers, although malignancies were usually detected on the right side, in the pericardium, or pulmonary arteries (*P*<0.001). Among malignant masses, 6 were entirely intramural. Compared with benign ones, malignant masses showed a greater diameter and were mainly inhomogeneous and infiltrating (*P*<0.001 for all); two-thirds of them were sessile (*P*=0.002), whereas about half were polylobate and accompanied by pericardial effusion (*P*<0.001). As for tissue characterization, interestingly, no significant difference in intensity at T1-weighted (both qualitative and quantitative) and FAT-SAT sequences were found between benign and malignant masses. Conversely, at T2-weighted-sequences, malignant masses were more frequently hyperintense compared with benign ones (*P*<0.001). At first-pass perfusion imaging, malignant masses showed intense, mainly heterogeneous enhancement compared with the benign ones, among which enhancement was present in half of the cases (*P*<0.001). At both EGE and LGE, malignant masses were more frequently hyperintense compared with benign ones (*P*<0.001 for both). No difference in heart chamber volumetric and functional assessment at cine sequences were observed between benign and malignant masses.

**Table 2.**
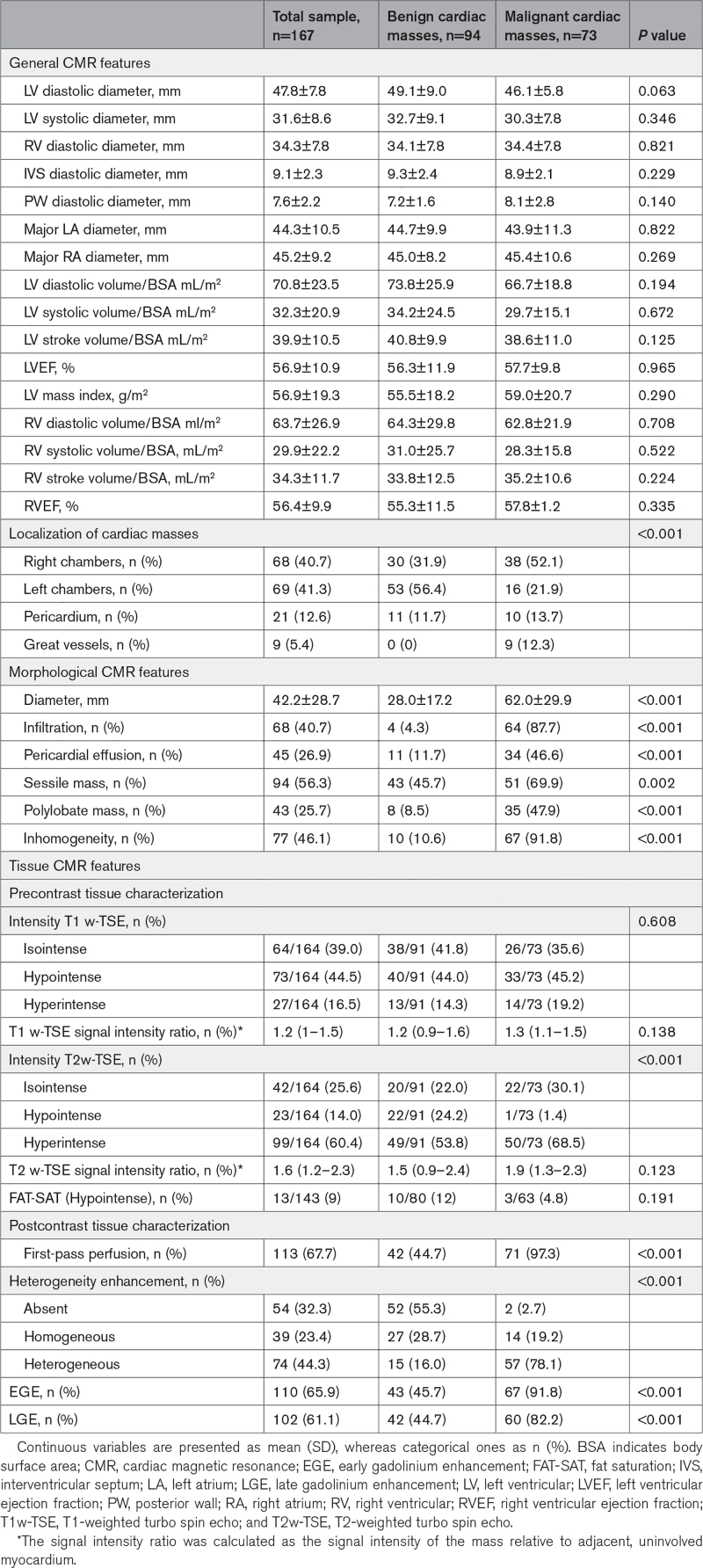
Comparison of CMR Morphological and Tissue Characteristics of Cardiac Benign and Malignant Masses

### CMR: A Multiparametric Approach to Define Malignancy

**Figure 2.**
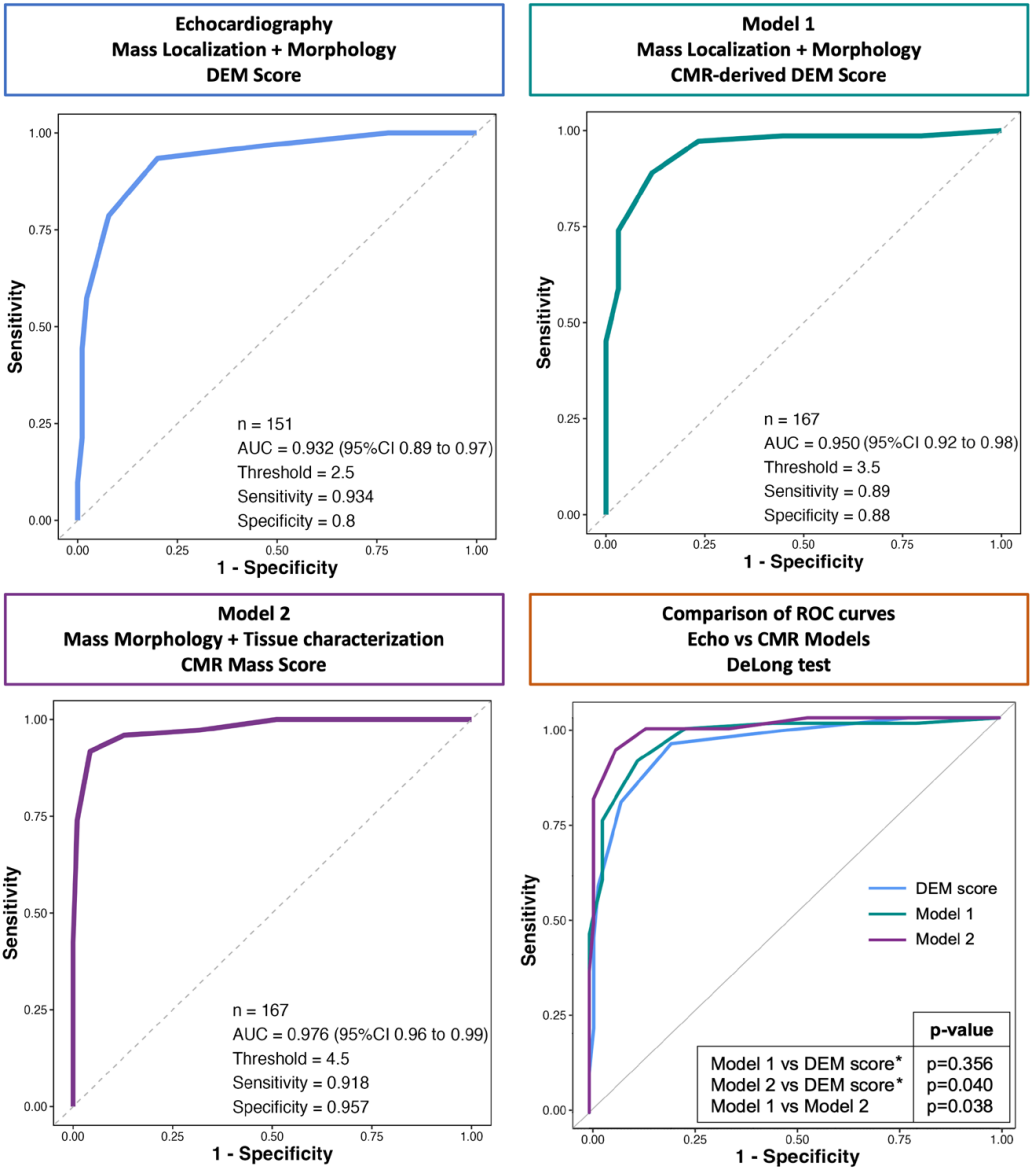
**Receiver operating characteristic (ROC) curves comparing the diagnostic accuracy of the diagnostic echocardiographic mass (DEM) score (echocardiography), the cardiac magnetic resonance (CMR)-derived DEM score (Model 1), and the CMR mass score (Model 2) in predicting cardiac masses malignancy.** *The comparisons between Models 1 and 2 and DEM score have been performed in the 151 patients in which echocardiography detected the presence of the cardiac mass.

Non-left localization, sessile, polylobate, inhomogeneity, infiltration, and pericardial effusion were confirmed to be independent predictors of malignancy in both univariate and multivariate models at CMR (Table 3A). Model 1, based on a CMR-derived assessment of mass localization and morphological features (as included in the DEM score) showed high accuracy in predicting malignancy, with an AUC, 0.950 (95% CI, 0.917–0.983), a sensitivity of 89%, specificity of 88% and accuracy of 87% (Figure 2).

**Table 3.**
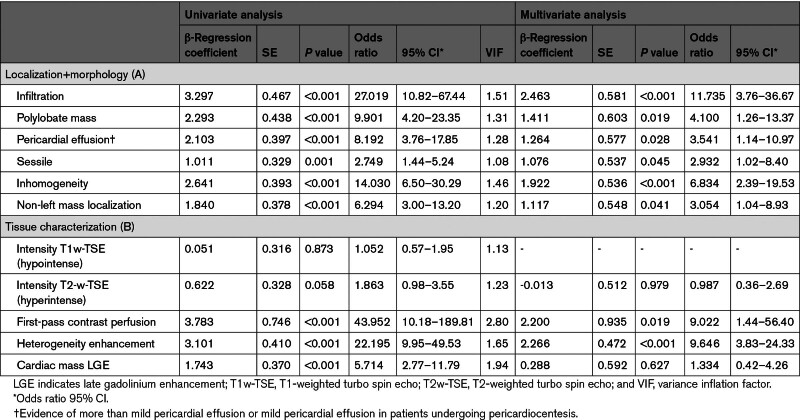
Univariate and Multivariate Logistic Regression Model Showing the Morphological and Tissue Characterization Variables Independently Associated With Malignancy

In a multivariate analysis including CMR-derived mass tissue characterization features, only first-pass contrast perfusion and heterogeneity enhancement were identified as independent predictors of malignant masses (Table 3A). The latter were then entered into a multivariate analysis, including mass localization, morphology, and tissue characterization, to derive a multiparametric model to predict malignancy. Sessile appearance, polylobate shape, pericardial effusion, infiltration, contrast perfusion, and heterogeneity enhancement were independent predictors of malignancy (Table 4). Based on the weight assigned to regression coefficients, a CMR-derived mass score (CMR mass score—Model 2) ranging from 0 to 8 was obtained from these 6 variables. Infiltration and first-pass contrast perfusion were assigned 2 points each, whereas pericardial effusion, sessile, polylobate shape, and heterogeneity enhancement were assigned 1 point each (Table S3). Model 2 showed the best accuracy in predicting malignancy, with an AUC of 0.976 (95% CI, 0.96–0.99), sensitivity of 92%, specificity of 96%, positive predictive value of 94%, negative predictive value of 94% and accuracy of 94% compared with Model 1 (*P* for comparison=0.038; Figure 2). The CMR mass score had the best AUC, which was significantly higher than each variable of the score taken individually (*P* for comparison<0.001 by DeLong test for all; Figure S2). A cutoff of ≥5 was obtained based on Youden index, which maximized sensitivity and specificity with respect to the histological diagnosis of malignancy (Figure 2; Table S4). The diagnostic accuracy of the CMR mass score was also confirmed in the study population excluding patients with LV thrombus (n=159 patients; Figure S3). Further details are shown in the Supplemental Material, Extended Results.

**Table 4.**
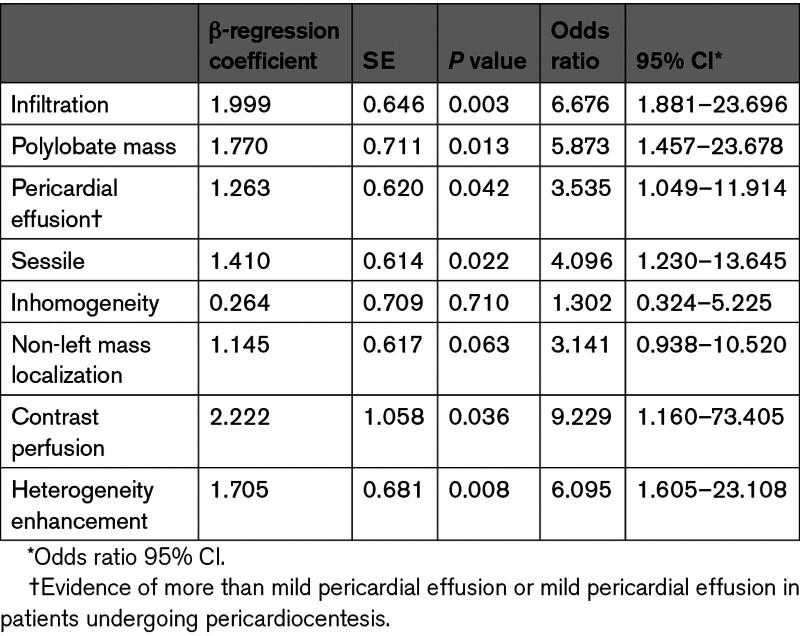
Multivariate Logistic Regression Model Showing the Morphological and Tissue Characterization Variables Independently Associated With Malignancy

### Interobserver Variability

Interobserver agreement expressed as Cohen κ was adequate (κ≥0.70) with a percentage of agreement >85% for all the parameters selected for the score and for the overall CMR mass score (Table S5). Further details are provided in Supplemental Material, Extended Results.

### CMR Versus Echocardiography: Diagnostic Accuracy

Compared with the echocardiographic and CMR-derived DEM score, the CMR mass score, integrating mass morphological and tissue characterization features, was more accurate in predicting CMs malignancy (*P*=0.040; Figure 2).

**Figure 3.**
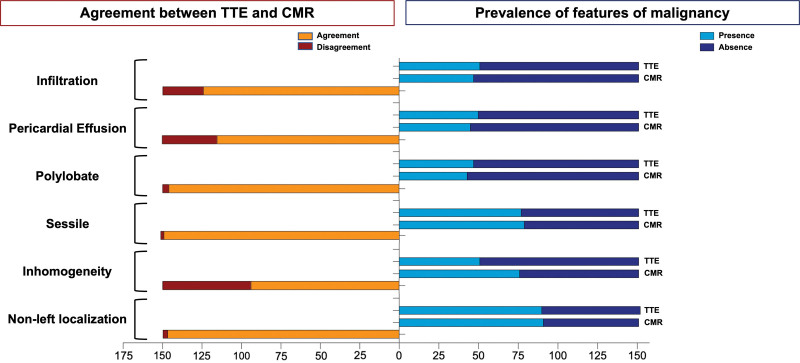
**Agreement between echocardiography and cardiac magnetic resonance (CMR) in the assessment of morphological features of cardiac masses.** TTE indicates transthoracic echocardiography.

Using histology as the gold standard, the diagnostic accuracy of the CMR mass score was superior to echocardiography, being overall 94% (versus 85% of the DEM score), with 92% sensitivity, 96% specificity (Table S6). The percentage agreement between the DEM score and CMR mass score assignment was 139/167 cases (83.2%), ranging from 78% to 87.2% for malignant and benign CMs (Table S6). The agreement expressed as Cohen κ for echocardiography versus CMR was κ=0.66, denoting substantial reliability between the 2 techniques. Interestingly, the number of malignant masses that were correctly identified by at least 1 between echo (DEM score, ≥3) and CMR (CMR mass score, ≥5) was 70/73 (96%) (Table S6). The 3 of 73 malignant masses misdiagnosed by both techniques were 2 metastases and 1 primary cardiac lymphoma. For each of the 6 variables identified as an independent predictor of malignancy, the rate of agreement between echocardiography and CMR is shown in Figure 3. The feature that presented the higher discrepancy in assignment between echocardiography and CMR was inhomogeneity, while localization, sessile, and polylobate appearance presented an almost complete agreement between the 2 imaging techniques (Figure 3). Interestingly, CMR showed infiltration in around 40% to 50% of patients, in which echocardiography did not detect it (Figure 3).

### Prognostic Value of the CMR Mass Score

The median follow-up of the overall population was 14 (6–28) months, specifically 15 (7–46) in patients with CMR mass score of <5 and 10 (5–22) in those with CMR mass score of ≥5. At follow-up, 54 (32.3%) patients died. As expected, most deaths were observed among patients with malignancies (42/54 patients, 77.8%). The prognostic value of the echocardiographic DEM score was confirmed (log-rank test,32.6, *P*<0.0001; hazard ratio, 5.50 [95% CI, 3.06–9.86]; Figure S4).

**Figure 4.**
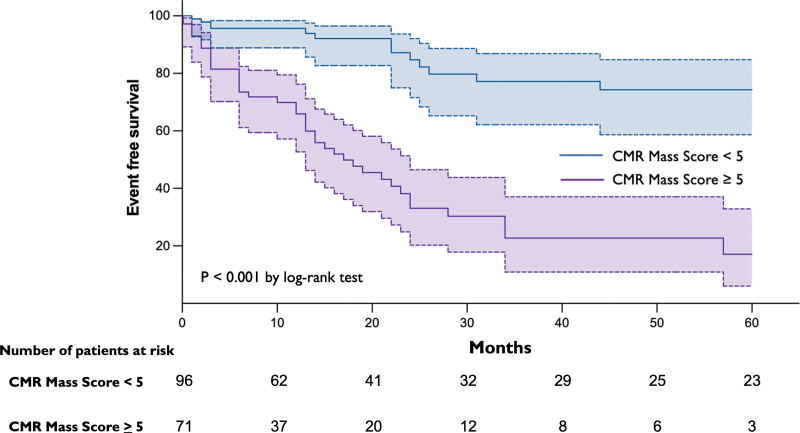
Kaplan-Meier survival estimates (all-cause death) for patients with <5 vs ≥5 cardiac magnetic resonance (CMR) features associated with malignancy.

The prognostic value of the CMR mass score (both as a dichotomic and continuous variable) was tested with Kaplan-Meier curve analysis. All-cause death was significantly higher for patients with a CMR mass score CMR of ≥5 compared with patients with a score of <5 (log-rank test, 39.8; *P*<0.0001; hazard ratio, 5.70 [95% CI, 3.264–9.942]; Figure 4). The CMR mass score also had a prognostic value when considered a continuous variable (hazard ratio, 1.39 [95% CI, 1.24–1.56]; *P*<0.001).

## DISCUSSION

In this study, we evaluated the CMR-derived features of CMs predictive of malignancy in a cohort of patients with a comprehensive echocardiogram performed at short-time interval from CMR, with histology as reference. A CMR-derived model (the CMR mass score), including mass morphology and tissue characterization to predict malignancy, was developed, tested for its accuracy compared with echocardiography and for its prognostic value. The main findings of this study are: (1) mass morphological features—non-left localization, sessile, polylobate, inhomogeneity, infiltration, and pericardial effusion—were confirmed to be independent predictors of malignancy when evaluated by CMR; (2) among CMs tissue characterization features, first-pass perfusion and heterogeneity enhancement were found to be independent predictors of malignancy; (3) the CMR mass score (range, 0–8 and cutoff, ≥5), including CMR-derived morphological and tissue characterization features (sessile appearance, polylobate shape, infiltration, pericardial effusion, first-pass contrast perfusion, and heterogeneity enhancement), showed an excellent accuracy to predict malignancy (AUC, 0.976 [95% CI, 0.96–0.99]), which was significantly higher compared with the DEM score and the CMR morphological model; (4) the agreement in the prediction of malignancy between the DEM score and the CMR mass score was good (κ=0.66), with inhomogeneity, pericardial effusion, and infiltration being the variables with higher discordance between the 2 techniques; (5) a CMR mass score of ≥5 predicted higher rate of all-cause death at follow-up.

The diagnosis of CMs remains challenging and benefits from stepwise multimodality imaging. In clinical practice, when approaching CMs, a 3-step CMR protocol is usually performed aiming to define: (1) mass localization, (2) mass morphological features, and (3) mass tissue characterization.^21,22^

### CMR: Mass Localization and Morphology

In our study, we confirmed that mass localization and morphology could accurately raise the suspicion of malignancy, also when evaluated at CMR. Considering only a morphological assessment and applying the previously validated DEM score in CMR, we showed that non-left side location, sessile and polylobate shape, inhomogeneous appearance, and presence of infiltration and pericardial effusion could per se predict malignancy with high diagnostic accuracy (AUC, 0.950 and accuracy, 87%).^9^ Notably, among the morphological features, mass size was not included in the malignancy prediction models, as even benign masses, for example, myxomas, can reach large dimensions, remaining asymptomatic for a long time.^23^ Moreover, it is not always possible to measure CMs size (eg, in case of diffusely infiltrating or polylobate masses, as shown in Figure 1), and there is not standardized reference for measurements in cases that could be measured. Second, the weight of pericardial effusion to predict malignancy at CMR is less marked compared with echocardiography, considering that in case of malignant masses presenting with clinically significant pericardial effusion, CMR is usually performed after pericardiocentesis. To account for this, the variable pericardial effusion included in the model of the CMR mass score was defined as more than mild pericardial effusion or mild pericardial effusion in patients undergoing pericardiocentesis. This might also explain the discrepancy in the detection of pericardial effusion between echocardiography and CMR. Nonetheless, the accuracy of the CMR-derived morphological model was high and comparable to the DEM score, highlighting the importance of a multiparametric approach that accurately predicts malignancy.

### CMR: Mass Tissue Characterization

Compared with echocardiography, CMR provides the relevant additional value of tissue characterization. The CMR mass score, including CMR-derived morphological and tissue characterization features, showed an excellent accuracy in predicting malignancy (AUC, 0.976), which was significantly higher compared with the echocardiographic and to the morphological model (versus DEM score, *P*=0.040; versus CMR-derived DEM score; *P*=0.038). Among tissue characterization features, it is remarkable that T1- and T2-weighted-intensity per se was not an independent predictor of malignancy. This might be explained by the rather subjective interpretation of this finding and by the heterogeneity of CMs. Thus, the role of T1 or T2 mapping in predicting malignancy needs to be evaluated in larger cohorts. Indeed, it is only recently that it has been suggested to add parametric mapping in the CMR protocol for patients with CMs,^24^ and its diagnostic and prognostic relevance still needs further investigation. On the other side, the CMR mass score included features that could be easily assessable, for which just the presence/absence needs to be assessed, minimizing the intra operator and interoperator variability related to the subjective interpretation of intensity. Indeed, we showed an adequate interoperator agreement in assessing all the parameters selected for the score and for the overall CMR mass score. These findings might be clinically relevant in setting up a standardized CMR protocol for CMs evaluation to be implemented in daily clinical practice, optimizing time and resources (in the common clinical scenario of limited availability of CMR slots, restricted time for each exam, and long waiting lists).

### CMs: CMR Versus Echocardiography

Compared with prior studies, for the first time, we directly compared echocardiography and CMR in a large cohort of patients with CMs, with histology as the gold standard. The CMR-derived morphological model did not significantly increase the diagnostic accuracy of the echocardiographic assessment (Figure 2; *P*=0.356); only the CMR mass score, integrating both mass morphological and tissue characterization features, was more accurate in predicting CMs malignancy compared with DEM score (echocardiography alone). This further confirms the additional value of the integration of mass morphology and tissue characterization features, emphasizing that CMR should not be considered simply as a super echo in the setting of CMs. Of interest, the agreement between echocardiography and CMR was κ=0.66, denoting substantial reliability between the 2 techniques. Among the morphological features with the higher discrepancy between echocardiography and CMR, CMR-detected infiltration in ≈40% to 50% of patients, in which echocardiography did not detect it. This could be due to the higher spatial resolution with better identification of the right chambers’ thickness (most involved in infiltration), better detection of contiguous structures and definition of their relationship.

### Clinical Implications

The number of malignant masses that were correctly identified by at least one between echo (DEM score, ≥3) and CMR (CMR mass score, ≥5) was 70/73 (96%), with a sensitivity and negative predictive value of 96%. These data provide useful information to improve the current flow chart recommended by the European Society of Cardiology guidelines for CMs assessment.^25^ Indeed, the different noninvasive investigations should not be considered on the same level,” as interchangeable, but they should be hierarchically performed, starting from echocardiography (I-level examination), moving on to CMR (noninvasive gold standard), reaching with these 2 techniques an almost perfect agreement compared with histology.^25^ In case of inconclusive results or CMR not available, cardiac computed tomography and positron emission tomography should be considered.^1^ Overall, with an almost perfect prediction of CMs malignancy with noninvasive multimodality imaging, the role and timing of CMs biopsies in the diagnostic work-up of CMs might be revised. Nonetheless, it should be stated that, so far, it is not possible to identify the precise histotype of CMs at CMR, although some histotypes might exhibit a typical CMR pattern.^12,22^

Finally, the CMR mass score provided crucial prognostic information. As expected, an increase in the cumulative score was associated with a progressively higher risk of death, being significantly higher in patients with at least 5 points.

### Study Limitations

Despite being the largest registry of CMs with echocardiography, CMR, and histological documentation, our study should be interpreted considering some limitations. First, the study was conducted in a single Institution. Secondly, surgical techniques, diagnostic procedures, and oncological management evolved during the recruitment period, thus, possibly affecting CMs’ identification and patients’ survival. Third, the prevalence of malignant CMs might have been underestimated; in fact, patients with advanced cancer often do not undergo further diagnostic investigations, thus, precluding a histopathologic confirmation. Fourth, in the current analysis, we did not include: (1) transesophageal echocardiography, contrast echocardiography, and nuclear imaging, because they are still not widely and promptly available, depending on local resources and operators’ expertise; (2) T1 and T2 mapping, because it was not available in all patients. Finally, no subgroup analysis for primary and secondary cardiac tumors was performed.

### Conclusions

A CMR-derived model, including mass morphology and tissue characterization, showed excellent accuracy, superior to echocardiography, in predicting CMs’ malignancy, with prognostic implications. Multimodality imaging, including at least echocardiography and CMR, should be recommended in the diagnostic work-up of CMs.

## ARTICLE INFORMATION

### Acknowledgments

Drs Paolisso, Bergamaschi, and Belmonte contributed conception and design of the study; Drs Angeli, Foà, Armillotta, Sansonetti, Fedele, Canton, Bodega, Amicone, and Suma organized the database and collected data; Drs Gallinoro, Bergamaschi, Belmonte, and PR performed the statistical analysis; Drs Niro and Lovato reviewed all the CMR images. Drs Paolisso, Bergamaschi, and Belmonte wrote the first draft of the article; Drs Niro, Pizzi, and Pavon wrote sections of the article. Drs Lovato, Carugo, Musthaq, Baggiano, Pavon, Guglielmo, Conte, Andreini, Pontone, and Pizzi revised the article and approved the final version of the article. All authors contributed to the article revision, read, and approved the submitted version. Dr Pizzi is the guarantor of the research and, as such, has full access to all the data in the study and takes responsibility for the integrity of the data and the accuracy of the data analysis.

### Sources of Funding

None.

### Disclosures

None.

### Supplemental Material

Supplemental Methods

Supplemental Results

Tables S1–S6

Figures S1–S4

References 26–34

## Supplementary Material

## References

[1] D’Angelo EC Paolisso P Vitale G Foà A Bergamaschi L Magnani I Saturi G Rinaldi A Toniolo S Renzulli M. Diagnostic accuracy of cardiac computed tomography and 18-f fluorodeoxyglucose positron emission tomography in cardiac masses. JACC Cardiovasc Imaging. 2020;13:2400–2411. doi: 10.1016/j.jcmg.2020.03.021 32563654 10.1016/j.jcmg.2020.03.021

[2] Lopez-Mattei JC Lu Y. Multimodality imaging in cardiac masses: to standardize recommendations, the time is now! JACC Cardiovasc Imaging. 2020;13:2412–2414. doi: 10.1016/j.jcmg.2020.04.009 32563655 10.1016/j.jcmg.2020.04.009

[3] Tyebally S Chen D Bhattacharyya S Mughrabi A Hussain Z Manisty C Westwood M Ghosh AK Guha A. Cardiac tumors: JACC cardiooncology state-of-the-art review. JACC CardioOncol. 2020;2:293–311. doi: 10.1016/j.jaccao.2020.05.009 34396236 10.1016/j.jaccao.2020.05.009 PMC8352246

[4] Rahouma M Arisha MJ Elmously A El-Sayed Ahmed MM Spadaccio C Mehta K Baudo M Kamel M Mansor E Ruan Y. Cardiac tumors prevalence and mortality: a systematic review and meta-analysis. Int J Surg. 2020;76:178–189. doi: 10.1016/j.ijsu.2020.02.039 32169566 10.1016/j.ijsu.2020.02.039

[5] Sultan I Bianco V Habertheuer A Kilic A Gleason TG Aranda-Michel E Harinstein ME Martinez-Meehan D Arnaoutakis G Okusanya O. Long-term outcomes of primary cardiac malignancies: multi-institutional results from the national cancer database. J Am Coll Cardiol. 2020;75:2338–2347. doi: 10.1016/j.jacc.2020.03.041 32381166 10.1016/j.jacc.2020.03.041

[6] Basso C Rizzo S Valente M Thiene G. Cardiac masses and tumours. Heart. 2016;102:1230–1245. doi: 10.1136/heartjnl-2014-306364 27277840 10.1136/heartjnl-2014-306364

[7] Urbini M Astolfi A Indio V Nannini M Pizzi C Paolisso P Tarantino G Pantaleo MA Saponara M. Genetic aberrations and molecular biology of cardiac sarcoma. Ther Adv Med Oncol. 2020;12:1758835920918492. doi: 10.1177/1758835920918492 10.1177/1758835920918492 PMC7238448 32489430

[8] Mankad R Herrmann J. Cardiac tumors: echo assessment. Echo Res Pract. 2016;3:R65–R77. doi: 10.1530/ERP-16-0035 27600455 10.1530/ERP-16-0035 PMC5292983

[9] Paolisso P Foà A Magnani I Bergamaschi L Graziosi M Angeli F Chiti C Fabrizio M Rinaldi A Stefanizzi A. Development and validation of a diagnostic echocardiographic mass score in the approach to cardiac masses. JACC Cardiovasc Imaging. 2022;15:2010–2012. doi: 10.1016/j.jcmg.2022.06.005 36357143 10.1016/j.jcmg.2022.06.005

[10] Paolisso P Foà A Bergamaschi L Graziosi M Rinaldi A Magnani I Angeli F Stefanizzi A Armillotta M Sansonetti A. Echocardiographic markers in the diagnosis of cardiac masses. J Am Soc Echocardiogr. 2023;36:464–473.e2. doi: 10.1016/j.echo.2022.12.022 36610495 10.1016/j.echo.2022.12.022

[11] Beroukhim RS Ghelani S Ashwath R Balasubramanian S Biko DM Buddhe S Campbell MJ Cross R Festa P Griffin L. Accuracy of cardiac magnetic resonance imaging in diagnosing pediatric cardiac masses: a multicenter study. JACC Cardiovasc Imaging. 2022;15:1391–1405. doi: 10.1016/j.jcmg.2021.07.010 34419404 10.1016/j.jcmg.2021.07.010 PMC11240235

[12] Fussen S De Boeck BW Zellweger MJ Bremerich J Goetschalckx K Zuber M Buser PT. Cardiovascular magnetic resonance imaging for diagnosis and clinical management of suspected cardiac masses and tumours. Eur Heart J. 2011;32:1551–1560. doi: 10.1093/eurheartj/ehr104 21498848 10.1093/eurheartj/ehr104

[13] Zhu D Yin S Cheng W Luo Y Yang D Lin K An Q Sun J Chen Y. Cardiac MRI-based multi-modality imaging in clinical decision-making: preliminary assessment of a management algorithm for patients with suspected cardiac mass. Int J Cardiol. 2016;203:474–481. doi: 10.1016/j.ijcard.2015.09.021 26551882 10.1016/j.ijcard.2015.09.021

[14] Pazos-López P Pozo E Siqueira ME García-Lunar I Cham M Jacobi A Macaluso F Fuster V Narula J Sanz J. Value of CMR for the differential diagnosis of cardiac masses. JACC Cardiovasc Imaging. 2014;7:896–905. doi: 10.1016/j.jcmg.2014.05.009 25129516 10.1016/j.jcmg.2014.05.009

[15] Shenoy C Grizzard JD Shah DJ Kassi M Reardon MJ Zagurovskaya M Kim HW Parker MA Kim RJ. Cardiovascular magnetic resonance imaging in suspected cardiac tumour: a multicentre outcomes study. Eur Heart J. 2021;43:71–80. doi: 10.1093/eurheartj/ehab635 34545397 10.1093/eurheartj/ehab635 PMC8720142

[16] Burke A Tavora FT. 2015 WHO classification of tumors of the heart and pericardium. J Thorac Oncol. 2016;11:441–452. doi: 10.1016/j.jtho.2015.11.009 26725181 10.1016/j.jtho.2015.11.009

[17] Pepi M Evangelista A Nihoyannopoulos P Flachskampf FA Athanassopoulos G Colonna P Habib G Ringelstein EB Sicari R Zamorano JL; European Association of Echocardiography. Recommendations for echocardiography use in the diagnosis and management of cardiac sources of embolism: European Association of Echocardiography (EAE) (a registered branch of the ESC). Eur J Echocardiogr. 2010;11:461–476. doi: 10.1093/ejechocard/jeq045 20702884 10.1093/ejechocard/jeq045

[18] Celeste F Muratori M Mapelli M Pepi M. The evolving role and use of echocardiography in the evaluation of cardiac source of embolism. J Cardiovasc Echogr. 2017;27:33–44. doi: 10.4103/jcecho.jcecho_1_17 28465991 10.4103/jcecho.jcecho_1_17 PMC5412748

[19] Mitchell C Rahko PS Blauwet LA Canaday B Finstuen JA Foster MC Horton K Ogunyankin KO Palma RA Velazquez EJ. Guidelines for performing a comprehensive transthoracic echocardiographic examination in adults: recommendations from the American society of echocardiography. J Am Soc Echocardiogr. 2019;32:1–64. doi: 10.1016/j.echo.2018.06.004 30282592 10.1016/j.echo.2018.06.004

[20] Lang RM Badano LP Mor-Avi V Afilalo J Armstrong A Ernande L Flachskampf FA Foster E Goldstein SA Kuznetsova T. Recommendations for cardiac chamber quantification by echocardiography in adults: an update from the American Society of Echocardiography and the European Association of Cardiovascular Imaging. J Am Soc Echocardiogr. 2015;28:1–39.e14. doi: 10.1016/j.echo.2014.10.003 25559473 10.1016/j.echo.2014.10.003

[21] Motwani M Kidambi A Herzog BA Uddin A Greenwood JP Plein S. MR imaging of cardiac tumors and masses: a review of methods and clinical applications. Radiology. 2013;268:26–43. doi: 10.1148/radiol.13121239 23793590 10.1148/radiol.13121239

[22] Gatti M D’Angelo T Muscogiuri G Dell’aversana S Andreis A Carisio A Darvizeh F Tore D Pontone G Faletti R. Cardiovascular magnetic resonance of cardiac tumors and masses. World J Cardiol. 2021;13:628–649. doi: 10.4330/wjc.v13.i11.628 34909128 10.4330/wjc.v13.i11.628 PMC8641001

[23] Foà A Paolisso P Bergamaschi L Rucci P Di Marco L Pacini D Leone O Galié N Pizzi C. Clues and pitfalls in the diagnostic approach to cardiac masses: are pseudo-tumours truly benign? Eur J Prev Cardiol. 2022;29:e102–e104. doi: 10.1093/eurjpc/zwab032 33655300 10.1093/eurjpc/zwab032

[24] Bonnes J Brink M Nijveldt R. How to evaluate cardiac masses by cardiovascular magnetic resonance parametric mapping? Eur Heart J Cardiovasc Imaging. 2023;24:1605–1607. doi: 10.1093/ehjci/jead221 37650512 10.1093/ehjci/jead221 PMC10667026

[25] Lyon AR López-Fernández T Couch LS Asteggiano R Aznar MC Bergler-Klein J Boriani G Cardinale D Cordoba R Cosyns B; ESC Scientific Document Group. 2022 ESC Guidelines on cardio-oncology developed in collaboration with the European Hematology Association (EHA), the European Society for Therapeutic Radiology and Oncology (ESTRO) and the International Cardio-Oncology Society (IC-OS). Eur Heart J. 2022;43:4229–4361. doi: 10.1093/eurheartj/ehac244 36017568 10.1093/eurheartj/ehac244

[26] Trojani M Contesso G Coindre JM Rouesse J Bui NB de Mascarel A Goussot JF David M Bonichon F Lagarde C. Soft-tissue sarcomas of adults; study of pathological prognostic variables and definition of a histopathological grading system. Int J Cancer. 1984;33:37–42. doi: 10.1002/ijc.2910330108 6693192 10.1002/ijc.2910330108

[27] Coindre JM Trojani M Contesso G David M Rouesse J Bui NB Bodaert A De Mascarel I De Mascarel A Goussot JF. Reproducibility of a histopathologic grading system for adult soft tissue sarcoma. Cancer. 1986;58:306–309. doi: 10.1002/1097-0142(19860715)58:2<306::aid-cncr2820580216>3.0.co;2-7 3719523 10.1002/1097-0142(19860715)58:2<306::aid-cncr2820580216>3.0.co;2-7

[28] Miller DV Tazelaar HD. Cardiovascular pseudoneoplasms. Arch Pathol Lab Med. 2010;134:362–368. doi: 10.5858/134.3.362 20196664 10.5858/134.3.362

[29] Habib G Lancellotti P Antunes MJ Bongiorni MG Casalta J-P Del Zotti F Dulgheru R El Khoury G Erba PA Iung B; ESC Scientific Document Group. 2015 ESC Guidelines for the management of infective endocarditis: the task force for the management of infective endocarditis of the European Society of Cardiology (ESC). endorsed by: European Association for Cardio-Thoracic Surgery (EACTS), the European Association of Nuclear Medicine (EANM). Eur Heart J. 2015;36:3075–3128. doi: 10.1093/eurheartj/ehv319 26320109 10.1093/eurheartj/ehv319

[30] Leone O Veinot JP Angelini A Baandrup UT Basso C Berry G Bruneval P Burke M Butany J Calabrese F. 2011 consensus statement on endomyocardial biopsy from the Association for European Cardiovascular Pathology and the Society for Cardiovascular Pathology. Cardiovasc Pathol. 2012;21:245–274. doi: 10.1016/j.carpath.2011.10.001 22137237 10.1016/j.carpath.2011.10.001

[31] Stewart S Winters GL Fishbein MC Tazelaar HD Kobashigawa J Abrams J Andersen CB Angelini A Berry GJ Burke MM. Revision of the 1990 working formulation for the standardization of nomenclature in the diagnosis of heart rejection. J Heart Lung Transplant. 2005;24:1710–1720. doi: 10.1016/j.healun.2005.03.019 16297770 10.1016/j.healun.2005.03.019

[32] Wei S Henderson-Jackson E Qian X Bui MM. Soft tissue tumor immunohistochemistry update: illustrative examples of diagnostic pearls to avoid pitfalls. Arch Pathol Lab Med. 2017;141:1072–1091. doi: 10.5858/arpa.2016-0417-RA 28745570 10.5858/arpa.2016-0417-RA PMC7864385

[33] Lestuzzi C Biasi S Nicolosi GL Lodeville D Pavan D Collazzo R Guindani A Zanuttini D. Secondary neoplastic infiltration of the myocardium diagnosed by two-dimensional echocardiography in seven cases with anatomic confirmation. J Am Coll Cardiol. 1987;9:439–445. doi: 10.1016/s0735-1097(87)80401-1 3805532 10.1016/s0735-1097(87)80401-1

[34] Nagueh SF Smiseth OA Appleton CP Byrd BF Dokainish H Edvardsen T Flachskampf FA Gillebert TC Klein AL Lancellotti P; Houston, Texas; Oslo, Norway; Phoenix, Arizona; Nashville, Tennessee; Hamilton, Ontario, Canada; Uppsala, Sweden; Ghent and Liège, Belgium; Cleveland, Ohio; Novara, Italy; Rochester, Minnesota; Bucharest, Romania; and St. Louis, Missouri. Recommendations for the evaluation of left ventricular diastolic function by echocardiography: an update from the American Society of Echocardiography and the European Association of Cardiovascular Imaging. Eur Heart J Cardiovasc Imaging. 2016;17:1321–1360. doi: 10.1093/ehjci/jew082 27422899 10.1093/ehjci/jew082

