# Supplementary material for: Cardiac Magnetic Resonance to Predict Cardiac Mass Malignancy: The CMR Mass Score
Source: Circ Cardiovasc Imaging. 2024 Mar 19;17(3):e016115. doi: 10.1161/CIRCIMAGING.123.016115 (PMC10949976; doi:10.1161/CIRCIMAGING.123.016115)
Supplement: Supplementary file 1 [file hci-17-e016115-s001.pdf]

Cardiac magnetic resonance (CMR) to predict cardiac mass malignancy: the CMR Mass Score

Authors: Pasquale Paolisso MD, PhD[a,b•], Luca, Bergamaschi MD[c•], Francesco Angeli MD[c], Marta Belmonte MD[b,d], Alberto Foà MD[c], Lisa Canton MD[c], Damiano Fedele MD[c], Matteo Armillotta MD[c], Angelo Sansonetti MD[c], Francesca Bodega MD[c], Sara Amicone MD[c], Nicole Suma MD[c], Emanuele Gallinoro MD, PhD[a], Domenico Attinà MD[c], Fabio Niro MD[c], Paola Rucci PhD[e], Elisa Gherbesi MD[f,g], Stefano Carugo MD[f,g], Saima Musthaq MD[h], Andrea Baggiano MD[g,h], Anna Giulia Pavon MD[i], Marco Guglielmo MD[j], Edoardo Conte MD[a], Daniele Andreini MD[a], PhD, Gianluca Pontone MD[g,k], Luigi Lovato MD[c*] and Carmine Pizzi MD[c*]

Affiliations:

(a) Clinical Cardiology and Cardiovascular Imaging Unit, Galeazzi-Sant'Ambrogio Hospital, IRCCS, Milan, Italy; Department of Biomedical and Clinical Sciences, University of Milan, Milan, Italy

(b) Department of Advanced Biomedical Sciences, University of Naples, Federico II, Naples, Italy

(c) Cardiology Unit, Cardiac Thoracic and Vascular Department, IRCCS Azienda Ospedaliera-Universitaria di Bologna; Department of Medical and Surgical Sciences – DIMEC; Alma Mater Studiorum, University of Bologna, Italy

(d) Cardiovascular Center Aalst, OLV Hospital, Aalst, Belgium

(e) Division of Hygiene and Biostatistics, Department of Biomedical and Neuromotor Sciences, Alma Mater Studiorum, University of Bologna, Bologna, Italy

(f) Department of Cardio-Thoracic-Vascular Diseases, Foundation IRCCS Ca' Granda Ospedale Maggiore Policlinico, Milan, Italy

(g) Department of Clinical Sciences and Community Health, University of Milan, Italy

(h) Department of Perioperative Cardiology and Cardiovascular Imaging, Centro Cardiologico Monzino IRCCS, Milan, Italy

(i) Division of Cardiology, Cardiocentro Ticino Institute, Ente Ospedaliero Cantonale, Via Tesserete, 48, 6900, Lugano, Switzerland

(j) Department of Cardiology, Division of Heart and Lungs, Utrecht University, Utrecht University Medical Center, Utrecht, The Netherlands

(k) Department of Biomedical, Surgical and Dentals Sciences, University of Milan, Milan, Italy

•The first two authors contributed equally to this work.

*The last two authors contributed equally to this work.

Corresponding author:

Carmine Pizzi, MD, FESC

Cardiology Unit, Cardiac Thoracic and Vascular Department, IRCCS Azienda Ospedaliera-Universitaria di Bologna. Department of Medical and Surgical Sciences – DIMEC

Alma Mater Studiorum, University of Bologna, Italy

Tel. +39 051 6364933

E-mail: carmine.pizzi@unibo.it

Supplemental Material

SUPPLEMENTAL METHODS

Definition and classifications of Cardiac Masses

All cases were classified according to the World Health Organization 2015 Classification of Tumors of the Heart and Pericardium (16); sarcomas were graded according to the Fédération Nationale des Centres de Lutte Contre le Cancer (FNCLCC) system (26,27). Pseudotumors, which represent an extremely heterogeneous group, were defined as lesions not originating from a neoplastic transformation of a specific cell type (28). Normal anatomical variants were excluded due to the inability to obtain a histological examination. Patients with suspected endocarditis underwent laboratory and imaging investigations according to guidelines (29). Only patients with CMs that did not meet the diagnostic criteria for infective endocarditis were included in our Registry.

Pathology

Surgical specimens. Gross evaluation and sampling of surgical specimens were performed following the standard indications for soft tissue tumors: masses up to 5 cm were entirely included; in those over 5 cm, one section per cm was sampled. From a paraffin-embedded block, 2-μm-thick sections were cut and routinely stained with Haematoxylin–Eosin.

Biopsy samples. Endomyocardial biopsies (EMB) and small surgical biopsies were rapidly fixed and processed by microwave. Following indications for transplant biopsy monitoring and the 2011 AECVP/SCVP Consensus Statement on EMB, serial sections were obtained, a part of which were stained using Haematoxylin–Eosin and the remainder left unstained for immunohistochemistry and special stainings (30).

Special stains included Alcian blue, Periodic acid-Schiff, Toluidine bleu, Von Kossa for calcium deposits, Mallory Trichrome for collagen, and Weigert-Van Gieson for elastic fibres, where appropriate.

According to tumor type appropriate immunohistochemistry panels were sequentially applied as described in the review by Wei et al (31,32).

Data collection and outcomes

For each patient, demographic and clinical data were collected. All patients underwent a complete diagnostic work-up, including clinical evaluation and laboratory testing (with specific examinations according to the clinical scenario). All patients were followed-up after the index presentation, and clinical data were obtained from outpatient visits or telephone interviews.

Echocardiography

All patients were evaluated by echocardiogram using a high-quality ultrasound machine (iE33, Affinity or Epiq 7C, Philips Healthcare, Eindhoven, The Netherlands). Echocardiographic examination was performed following the recommendations of the American Society of Echocardiography and the European Association of Cardiovascular Imaging with the patients in the left lateral recumbent position with the use of standard views (17-20). At least three consecutive beats were recorded for each view, and all images were stored for offline analysis (Intellispace, Philips Healthcare, Eindhoven, The Netherlands). The recorded images were analyzed offline by expert cardiologists with experience in cardiac imaging, blinded to clinical information and CMs histology. The cardiac imager analyzed all echoes using a pre-specified worksheet to mark the presence or absence of the following parameters: location (left/right, atrium/ventricle, pericardium, great vessels), site of attachment (interatrial/interventricular septum or roof/side wall of the atrium, ventricular free wall), dimension, shape (regular/irregular), margins (well defined/irregular - if more than 50% of the border was clearly demarcated), mass characteristics (sessile - attached directly by the base and not raised upon a stalk, pedunculated - raised upon a stalk - or polylobate - having two or more lobes), mobility, infiltration

[defined as disruption of neighboring tissue and extension of the mass across the pericardium into myocardium, with interruption of epicardial and endocardial contours or, alternatively, by the presence of at least one of the following echocardiographic features i) evidence of a different reflectivity compared to the normal myocardium as infiltrating masses usually have a peculiar, granular echocardiographic texture; ii) increased thickness in comparison with the adjacent myocardial segments; iii) hypo/akinesia of a focal myocardial area compared to closest cardiac segments in absence of coronary distribution that could lead to the suspicion of ischemic etiology] (33), pericardial effusion (defined as a fluid accumulation between the two pericardial layers) and echogenicity pattern (hypo-, iso- or hyperechogenic as compared with normal myocardium). Left ventricle end-diastolic diameter (LVEDD) and volume (LVEDV) were calculated according to the ASE and EACVI recommendations (19,20). LVEF was calculated using Simpson's biplane method. LV diastolic function was assessed as recommended by the ASE/EACVI Guidelines, including E wave, e' velocities, E/e', left atrial volume index (LAVi), and tricuspid regurgitation velocity (34).

Cardiac Magnetic Resonance

The ECG-gated balanced steady-state free precession (SSFP) pulse sequence for cine images were acquired in the four-chambers, two-chambers and three-chambers long-axis (LA); in addition, a full stack of short-axis (SA) views was acquired from base to the apex to provide full coverage of the left and right ventricles. The T1-weighted and the T2-weighted inversion recovery fast-spin echo (IR FSE) sequences, images were acquired in identical long- and short-axis views than the cine images. The T1-weighted were repeated with fat saturation pre-pulse, if needed. Following this, an intravenous bolus dose of 0.1 mmol/kg Gd-DTPA (Dimeglumine gadopentetate) was administered at a rate of 5 ml/s by an MRI-compatible power injector, followed by 30ml saline flush (5ml/s). The first-pass perfusion imaging was performed simultaneously with the injection of gadolinium. Immediately after first-pass perfusion

imaging, a second bolus dose of 0.1 mmol/kg Gd-DTPA was administered. EGE images were acquired 1-4 minutes after gadolinium injection with a fixed inversion time (TI) of 440 ms (inversion recovery-prepared T1-weighted gradient echo). 10 minutes after gadolinium injection, a 'Look Locker' sequence was performed to obtain the most appropriate TI to null the signal intensity of normal myocardium. LGE images were then acquired 10-15 minutes after gadolinium injection with identical pulse sequence parameters as for EGE apart from the specifically determined TI.

SUPPLEMENTAL RESULTS

Benign vs malignant masses: clinical presentation

Baseline characteristics, cardiovascular risk factors, comorbidities, and clinical presentation are reported in **Supplemental Table S1**. Age at presentation, body mass index, cardiovascular risk factors, and comorbidities were similar in the two groups. Patients with malignant masses present more frequently with dyspnea - mostly in NYHA Class III/IV - and with a lower rate of incidental diagnosis than benign ones ($p<0.001$). Moreover, patients with malignant masses exhibited a higher rate of pulmonary embolism ($p=0.008$). The echocardiographic characteristics of both groups are shown in **Supplementary Table S2**.

Sensitivity analysis

We tested the predictive accuracy of the CMR Mass Score in our study population, excluding patients with left ventricular thrombus. Among the 24 thrombi included, 8 were in the left ventricle (with the remaining ones being in the right ventricle or in the atrial chambers). Thus, the subset population without LV thrombus included 159 patients. In this subset population, the CMR Mass score was confirmed to have an excellent diagnostic performance in discriminating malignancy of cardiac masses (AUC 0.974, 95% CI 0.95-0.99, $p<0.001$), which was significantly higher compared to the CMR-derived DEM score (AUC 0.948, 95% CI 0.91-0.98, $p<0.001$; p-value for comparison 0.03 at DeLong test) (**Supplementary Figure S3**).

Inter-observer variability

All the 167 CMRs recorded were randomly selected and re-analyzed by a cardiologist in training (a young cardiologist who just completed a 1-year fellowship in cardiac imaging and was at the beginning of his/her career), after an appropriate training session, blinded to patient clinical data. Inter-observer

variability of the variables selected for the score (defined as proposed in the Methods Section and expressed as dichotomized data according to the presence or absence in each CMR) was estimated by Cohen's coefficient. Adequate agreement was defined as $\kappa \geq 0.70$. Inter-observer agreement expressed as Cohen's κ was adequate ($\kappa \geq 0.70$) with a percentage of agreement > 85% for all the parameters selected for the score and for the overall CMR Mass Score (**Supplementary Table S5**). In detail, inter-observer Cohen's κ was 0.72, 0.75, 0.87, 0.74, 0.86, and 0.80 for infiltration, polylobate mass, pericardial effusion, sessile, first-pass contrast perfusion, and heterogeneity enhancement respectively, indicating good or excellent reliability for all parameters.

SUPPLEMENTAL FIGURES

Supplemental Figure S1. Study flowchart.

*All patients (N=273 patients with cardiac masses, histology and good quality echo images) included in the study published in JACC Cardiovascular Imaging 2022 (PMID: 36357143) were part of the group of “patients with CMs, histology and echo” of the current paper (N=323). Thus, in the current paper, 50 additional patients were included, being recruited between January 2021 and December 2022.

**Among the 167 patients, 160 were also part of the population included in JASE 2023 (PMID: 36610495), and 7 were part of the 10 additional patients.

Supplemental Figure S2. Diagnostic accuracy of the CMR Mass Score in predicting CM’ malignancy compared to each variable taken individually.

Supplemental Figure S3. Diagnostic accuracy of the CMR-derived DEM score and CMR Mass Score in the study population excluding patients with left ventricular thrombus.

Supplemental Figure S4. Kaplan-Meier survival estimates (all-cause death) for patients with < 3 versus ≥3 DEM Score.

Supplemental Figure S1.

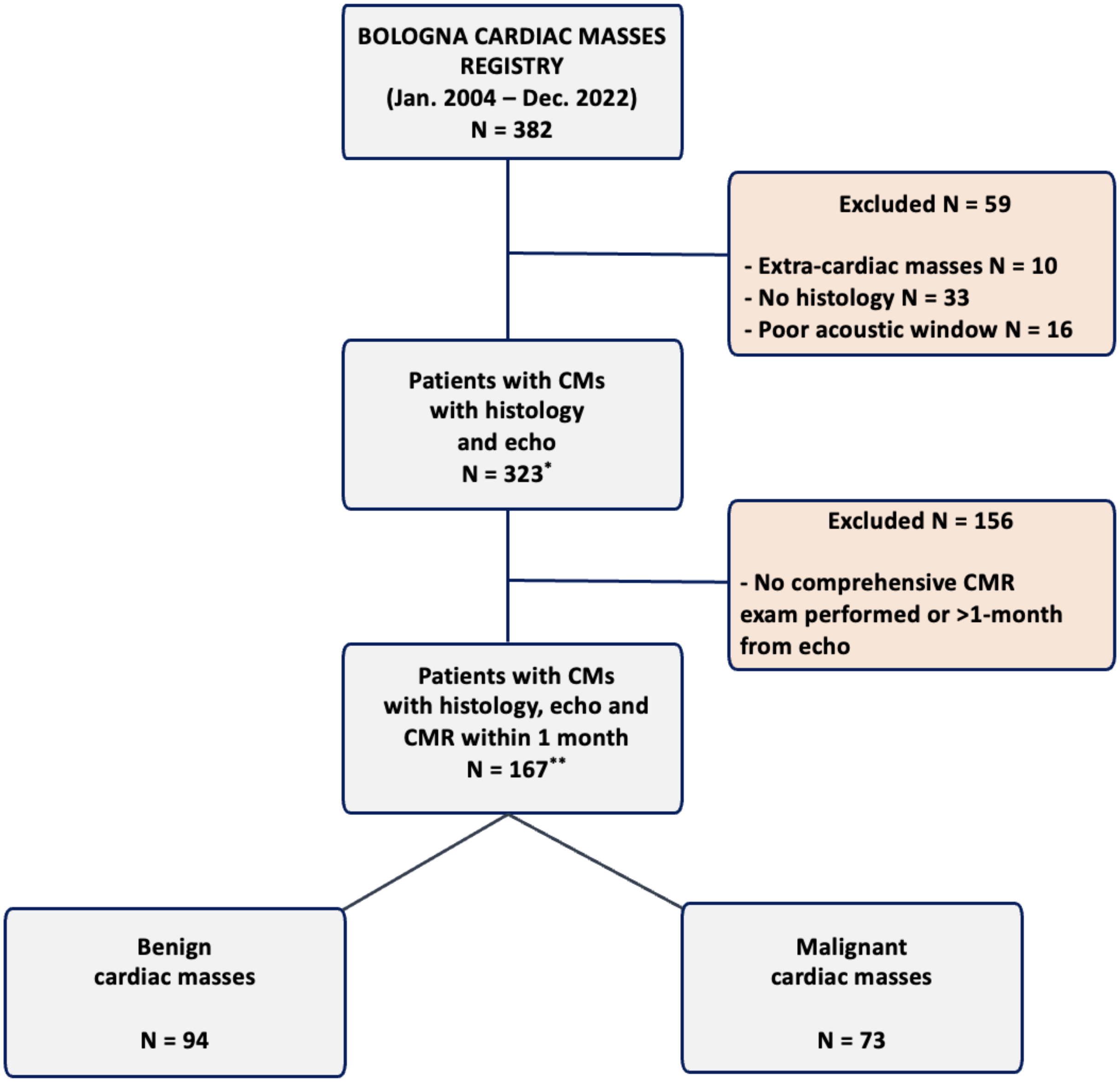

Supplemental Figure S2.

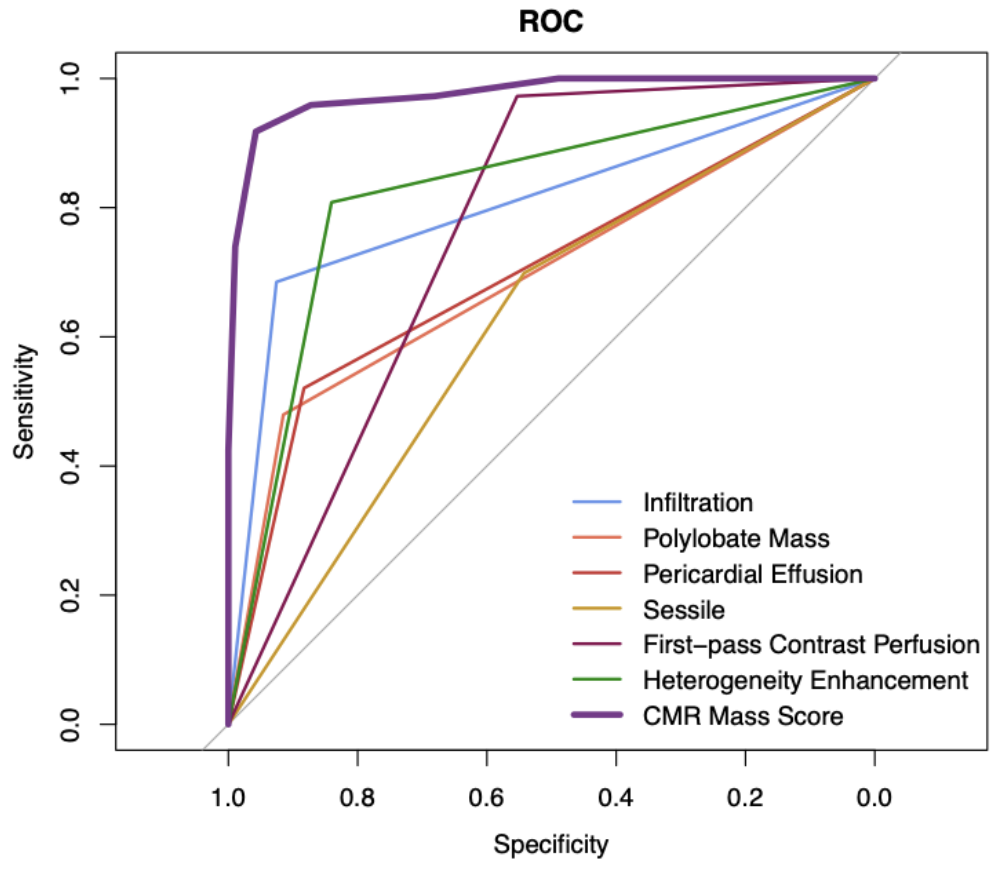

	AUC	p-value	95% CI
Infiltration	0.805	<0.001	0.74 – 0.86
Polylobate Mass	0.697	<0.001	0.63 – 0.76
Pericardial Effusion	0.702	<0.001	0.64 – 0.77
Sessile	0.621	0.006	0.55 – 0.69
First-pass Contrast Perfusion	0.763	<0.001	0.71 – 0.82
Heterogeneity Enhancement	0.824	<0.001	0.77 – 0.88
CMR Mass Score	0.976	<0.001	0.96 – 0.99

Supplemental Figure S3.

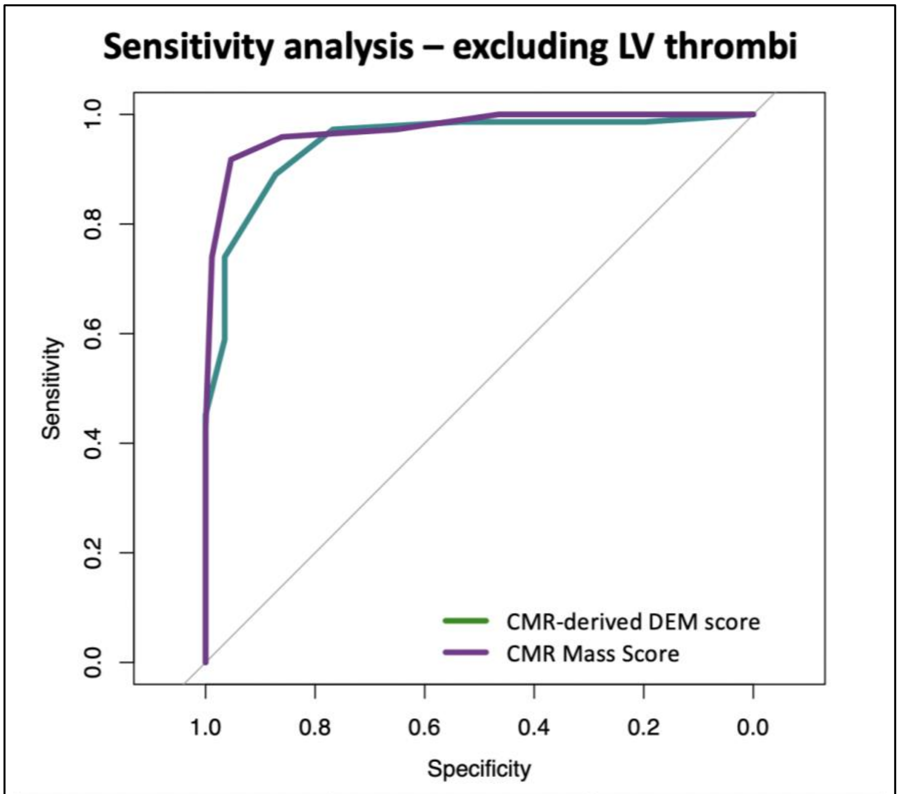

	AUC (95%CI)	p-value for comparison
CMR-derived DEM score	0.95 (0.91 - 0.98)	0.03
CMR Mass Score	0.97 (0.95 – 0.99)	

Supplemental Figure S4.

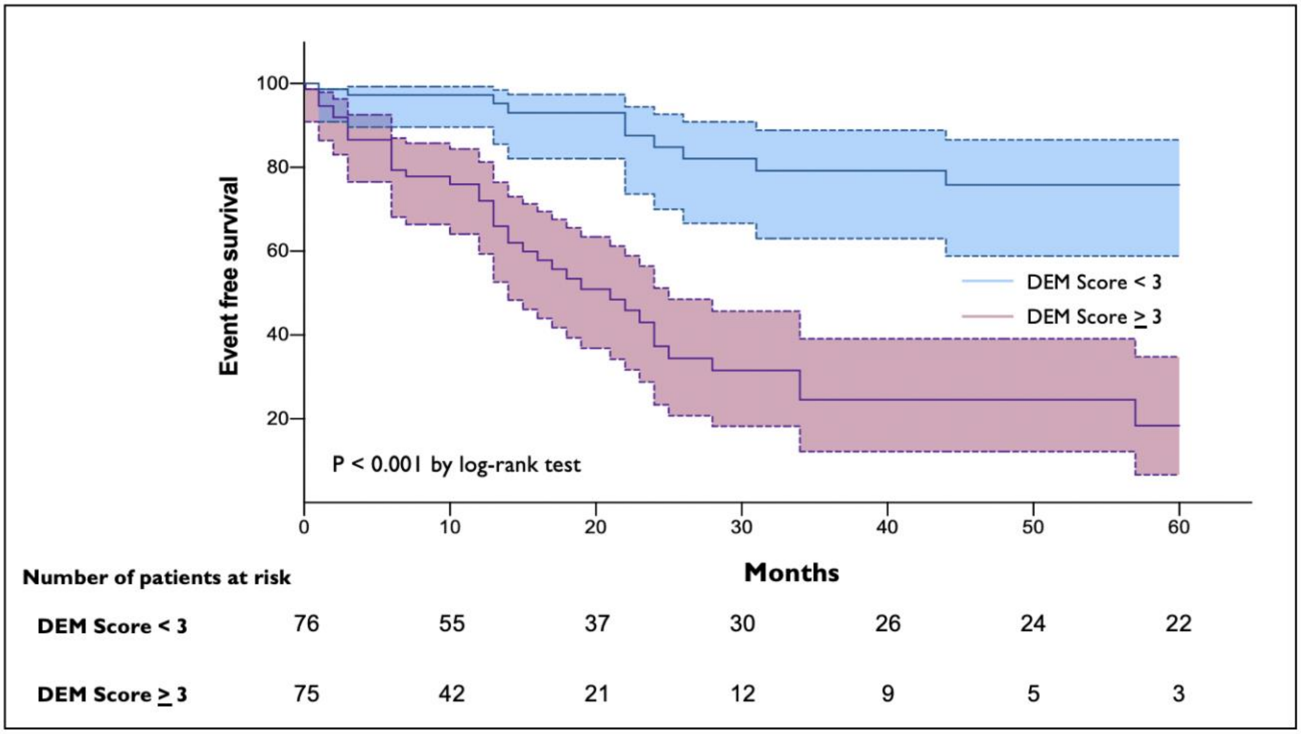

SUPPLEMENTAL TABLES

Supplemental Table S1. Baseline clinical and laboratory characteristics of study population stratified according to benign or malignant cardiac masses.

	Total sample<br>N = 167	Benign cardiac masses<br>N = 94	Malignant cardiac masses<br>N = 73	P-value
**Male gender, n (%)**	80 (47.9)	39 (41.5)	41 (56.2)	0.060
**Age, years**	59.2 ± 16.7	59 ±16.5	59.6 ± 17	0.773
**Age groups, n (%)**				0.682
21-40 years	33 (19.8)	19 (20.2)	14 (19.2)	
41-60 years	45 (26.9)	23 (24.5)	22 (30.1)	
61-80 years	78 (46.7)	47 (50)	31 (42.5)	
81-100 years	11 (6.6)	5 (5.3)	6 (8.2)	
**BMI, kg/m^2**	25.5 ± 4.2	25.4 ± 4.1	25.6 ± 4.5	0.618
**Cardiovascular risk factors**				
**Smoking habit, n (%)**	87 (52.1)	48 (51.1)	39 (53.4)	0.762
**Hypertension, n (%)**	88 (52.7)	52 (55.3)	36 (49.3)	0.441
**Dyslipidemia, n (%)**	67 (40.1)	40 (42.6)	27 (37)	0.467
**T2DM, n (%)**	25 (15)	17 (18.1)	8 (11)	0.201
**Medical History**				
**Congestive Heart failure, n (%)**	24 (14.4)	14 (14.9)	10 (13.7)	0.827
**Prior stroke, n (%)**	29 (17.4)	19 (20.2)	10 (13.7)	0.270
**History of neoplasia, n (%)**	67 (40.1)	33 (35.1)	34 (46.6)	0.134
**Clinical presentation**				
**Incidental diagnosis, n (%)**	80 (47.9)	63 (67)	17 (23.3)	<0.001
**Dyspnea, n (%)**	71 (42.5)	30 (31.9)	41 (56.2)	0.002
**NYHA classes, n (%)**				0.016
I- II	121 (72.5)	75 (79.8)	46 (63)	
III-IV	46 (27.5)	19 (20.2)	27 (37)	
**Chest pain, n (%)**	30 (18)	13 (13.8)	17 (23.3)	0.114
**Palpitations, n (%)**	21 (12.6)	13 (13.8)	8 (11)	0.579
**Peripheral embolism, n (%)**	15 (9)	10 (10.6)	5 (6.8)	0.396
**Pulmonary embolism, n (%)**	16 (9.6)	4 (4.3)	12 (16.4)	0.008
**Laboratory parameters**				
**Creatinine, mg/dL**	0.84 [0.70 - 1.02]	0.85 [0.72 - 1]	0.82 [0.70 - 1.04]	0.594

**GFR, mL/min**	85 [70 - 98]	85 [68 - 98]	88 [71 - 99]	0.751
**Hb, g/dL**	12.7 ± 1.9	13 ± 1.5	12.3 ± 2.2	0.031
**WBC, n/mmc**	7.855 [6.415-10.532]	7.605 [6.292-9.760]	8.885 [6.825-11.005]	0.155
**CRP, mg/dL**	1 [0.3 - 4.5]	0.8 [0.2 - 2]	3.1 [0.7 – 6.1]	0.001
**LDH, mg/dL**	236 [172 – 344]	210 [149 – 298]	290 [204 – 412]	0.001

Continuous variables are presented as mean (SD) or median (IQR) when appropriate; categorical ones as n (%). Abbreviations: BMI: body mass index; T2DM: Type 2 Diabetes Mellitus. NYHA: New York Heart Association; GFR: glomerular filtration rate; Hb: hemoglobin; WBC: white blood cells; CRP: C-reactive protein; LDH: lactate dehydrogenase.

Supplemental Table S2. Comparison of echocardiographic features between benign or malignant masses.

	Total sample N = 167	Benign cardiac masses N = 94	Malignant cardiac masses N = 73	P-value
**LVEDD, mm**	46.2 ± 6.6	47 ± 7.1	45.3 ± 5.7	0.711
**LVEDV, ml**	92.1 ± 38	97.3 ± 44.9	85.4 ± 25	0.224
**LVEF, %**	60 ± 10	59 ± 12	60 ± 9	0.709
**LAV, ml**	50 ± 6	51 ± 4	49 ± 6	0.323
**sysPAP, mmHg**	33 ± 15	30 ± 10	36 ± 11	0.364
**Mitral regurgitation, n (%)**				0.060
Mild, n (%)	92 (55.1)	57 (60.6)	35 (48)	
Moderate/severe, n (%)	21 (12.6)	13 (13.8)	8 (11)	
**Mitral stenosis, n (%)**				0.656
Mild, n (%)	1 (0.6)	1 (1.1)	0 (0)	
Moderate/severe, n (%)	4 (2.4)	2 (2.1)	2 (2.7)	
**Aortic regurgitation, n (%)**				0.273
Mild, n (%)	33 (19.8)	16 (17)	17 (23.3)	
Moderate/severe, n (%)	6 (3.6)	2 (2.1)	4 (5.5)	
**Aortic stenosis, n (%)**				0.064
Mild, n (%)	15 (9)	12 (12.8)	3 (4.1)	
Moderate/severe, n (%)	2 (1.2)	2 (2.1)	0 (0)	
**Tricuspid regurgitation, n (%)**				0.267
Mild, n (%)	102 (61.1)	59 (62.8)	43 (58.9)	
Moderate/severe, n (%)	14 (8.4)	5 (5.3)	9 (12.3)	
**m/s pericardial effusion, n (%)**	26 (15.6)	2 (2.1)	24 (32.9)	<0.001
**Infiltration, n (%)**	51 (30.5)	8 (8.5)	43 (58.9)	<0.001
**Implant**				<0.001
Lat/sup. Atrial wall, n (%)	40 (24)	19 (20.2)	21 (28.8)	
IAS, n (%)	41 (24.5)	36 (38.3)	5 (6.8)	
**Max. CM diameter, mm**	38.5 ± 21	29.4 ± 13	52 ± 23	<0.001
**Inhomogeneity, n (%)**	51 (30.5)	18 (19.1)	33 (45.2)	<0.001
**Irregular margins, n (%)**	48 (28.7)	16 (17)	32 (43.8)	0.001
**Mobility, n (%)**	49 (29.3)	40 (42.6)	9 (12.3)	<0.001
**Sessile mass, n (%)**	79 (47.3)	40 (42.6)	39 (53.4)	0.002
**Polylobate mass, n (%)**	47 (28.1)	12 (12.8)	35 (47.9)	<0.001

Continuous variables are presented as mean (SD), while categorical ones as n (%). Abbreviations: LVEDD: Left Ventricular End-Diastolic diameter; LVEDV: Left Ventricular End-Diastolic Volume; LVEF: Left Ventricular Ejection Fraction; LAV: Left Atrial Volume; sysPAP: systolic pulmonary artery pressure; m/s: moderate to severe; IAS: Inter-Atrial Septum; CM: Cardiac Mass.

Supplemental Table S3. CMR Mass Score development.

CMR-Mass Score - Multivariable analysis							
	Standard Error	p-value	Odds Ratio	95% CI*	β regression coefficient	Weight	Score
**Infiltration**	0.646	0.003	6.676	1.881 - 23.696	1.999	1.58	2
**Polylobate mass**	0.711	0.013	5.873	1.457 - 23.678	1.770	1.40	1
**Pericardial effusion**	0.620	0.042	3.535	1.049 - 11.914	1.263	1**	1
**Sessile**	0.614	0.022	4.096	1.230 – 13.645	1.410	1.12	1
**Contrast perfusion**	1.058	0.036	9.229	1.160 – 73.405	2.222	1.76	2
**Heterogeneity enhancement**	0.681	0.008	6.095	1.605 – 23.108	1.705	1.35	1

*Odds ratio 95% CI

**The regression coefficient of each of these variables was divided by the smallest coefficient in the model, which was taken as reference (weight of 1).

Supplemental Table S4. The accuracy indicators for each cut-off of the CMR Mass Score.

Cut-off	Sens.	Spec.	Youden's I
**0**	1.00	1.00	0.00
**1**	1.00	0.21	0.21
**2**	1.00	0.49	0.49
**3**	0.97	0.68	0.65
**4**	0.96	0.87	0.83
**5**	**0.92**	**0.96**	**0.88**
**6**	0.74	0.99	0.73
**7**	0.43	1	0.43
**8**	0.19	1	0.19

Abbreviations**:** Sens: Sensibility; Spec: Specificity.

Supplemental Table S5. Inter-observer variability in the assessment of the CMR Mass Score and its metrics taken individually.

	Infiltration	Polylobate Mass	Pericardial effusion	Sessile	First-pass contrast perfusion	Heterogeneity enhancement	CMR Mass Score $\geq$5
**N° of agreements**	145/167	149/167	158/167	146/167	155/167	150/167	151/167
**% of agreements**	86.8%	89.2%	94.6%	87.4%	92.8%	89.8%	90.4%
**Cohen's κ**	0.72	0.75	0.87	0.74	0.86	0.80	0.81

Supplemental Table S6. Comparison between echocardiography and cardiac magnetic resonance for assessment of malignancy in patients with cardiac masses.

	Total sample N = 167	N° of correct assignment/Total Benign CM N = 94	N° of correct assignment/Total Malignant CM N = 73	Sensitivity	Specificity	PPV	NPV	Accuracy
**DEM score ≥ 3* vs Histology**	129/151 (85.4%)	72/90 (80%)	57/61 (93.4%)	93%	80%	76%	95%	85%
**DEM score ≥ 3 vs Histology**	129/167 (77%)	72/94 (76.6%)	57/73 (78%)	78%	77%	72%	82%	77%
**CMR Mass score ≥ 5 vs Histology**	157/167 (94%)	90/94 (95.7%)	67/73 (92%)	92%	96%	94%	94%	94%
**DEM Score ≥ 3 vs CMR Mass score ≥ 5**	139/167 (83.2%)	82/94 (87.2%)	57/73 (78%)	79%	87%	83%	85%	84%
**DEM Score ≥ 3 and/or CMR Mass score ≥ 5 vs Histology**	154/167 (92.2%)	84/94 (89.4%)	70/73 (96%)	96%	89%	88%	97%	92%

*These percentages are calculated only considering the cardiac masses detected by echo n = 151.

Abbreviations: CM: cardiac mass; PPV: Positive Predictive Value; NPV: Negative Predictive Value; CMR: cardiac magnetic resonance; DEM: diagnostic echocardiographic mass score.
